# Enhancing the *in vitro* architecture of human disease

**DOI:** 10.1242/dmm.053091

**Published:** 2026-07-09

**Authors:** Kirsty Hooper, Dina Mikimoto, Vivian Li, Austin Smith, Joseph Wu

**Affiliations:** ^1^ The Company of Biologists, Bidder Building, Station Road, Histon, Cambridge CB24 9LF, UK; ^2^ Stem Cell and Cancer Biology Laboratory, The Francis Crick Institute, London NW1 1AT, UK; ^3^ Living Systems Institute, University of Exeter, Exeter EX4 4QD, UK; ^4^ Stanford Cardiovascular Institute, Stanford University, Stanford, CA 94305, USA; ^5^ Department of Medicine (Division of Cardiovascular Medicine), Stanford University, Stanford, CA 94305, USA

## Abstract

**Summary:** This Editorial introduces DMM's new Special Issue on ‘*In Vitro* Models of Human Disease to Inform Mechanism and Drug Discovery'. The Guest Editors discuss how such models further our understanding of the mechanisms of disease, accelerating drug development and enabling customised approaches to treatment.

**Figure:**
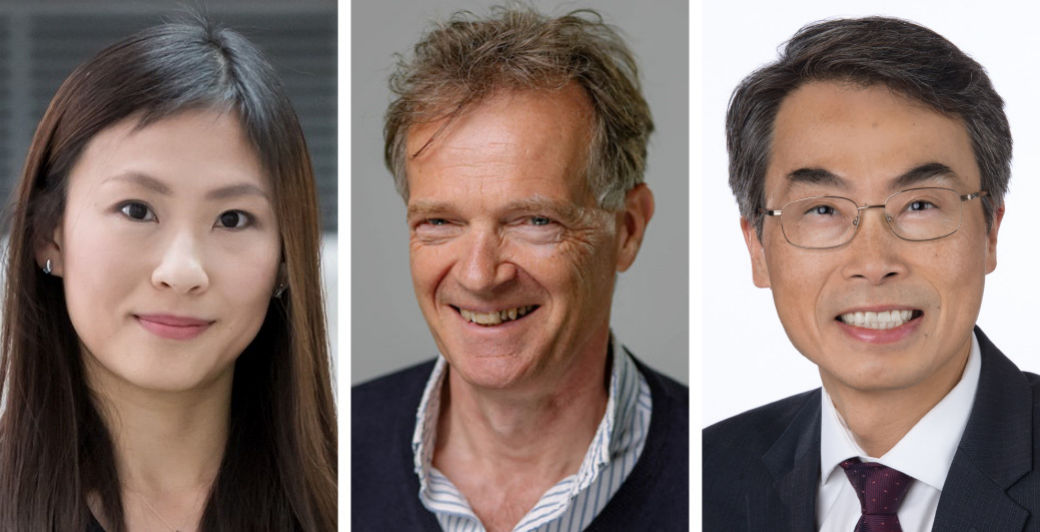
Editors Vivian Li, Austin Smith and Joseph Wu (left to right)

During the past decade, there has been an surge in the emergence of innovative *in vitro* disease models that are reshaping our understanding of disease mechanisms and therapeutic development. Advanced induced pluripotent stem cell (iPSC) cultures, organoids, assembloids and even organ-on-a-chip technologies are all exploiting scientific progress in biomaterials, stem cell biology and tissue engineering to build human-cell-based models that are not only more physiologically representative but also more predictive. These systems bridge the gap between traditional cell culture and whole-organ systems by incorporating cellular diversity, spatial organisation, microenvironmental niches and tissue architecture. With international regulatory bodies further encouraging the development of reliable *in vitro* systems for biomedical research (Bogani et al., 2026), with the ultimate aim of reducing and refining animal use in research and industry, these models have gained even more momentum.

To continue our support of this field, we launched a special issue of DMM focused on *In Vitro* Models of Human Disease to Inform Mechanism and Drug Discovery, which is guest edited by Joseph Wu, Austin Smith and DMM Editor Vivian Li. The goal in shaping this Special Issue was to compile original Research, Resources & Methods and Review-type articles that showcase the latest advances in *in vitro* model systems and highlight how these approaches can deepen our understanding of disease mechanisms while accelerating drug discovery and therapeutic development.

Importantly, the Special Issue also highlights how these advanced *in vitro* systems can work in concert with animal models to build a holistic view of human disease.

## Complementarity between *in vitro* and *in vivo* modelling

Rysankova et al. (2026) explore synergy between *in vitro* and *in vivo* modelling for disease research by establishing an iPSC line from a transgenic minipig model of Huntington's disease (HD). Interestingly, before hallmark pathological features appear in the minipig model, iPSCs derived from the HD animals already exhibit altered metabolic profiles, indicating that pathology may begin at a much earlier cellular stage than previously appreciated. By deriving iPSCs from an animal model, this study established an *in vitro* model that can be directly integrated with the corresponding *in vivo* model and used to investigate early disease mechanisms that precede overt phenotype in model organisms.

Human iPSC-based models can also complement findings in animal models of the same disease by providing a human-relevant platform for disease investigation. West et al. (2026) used human trisomy 21 iPSC-derived neural cells to model trisomy 21 and test Leucettinib-21, an inhibitor of DYRK1A that is elevated in Down syndrome, and other neurodevelopmental and neurodegenerative diseases. The authors show that Leucettinib-21 decreases activity of DYRK1A in human trisomy 21 iPSC-derived cultures, which supports findings in animal models of Down syndrome and encourages future clinical trials with this drug.

Continuing the theme of synergy between *in vitro* and *in vivo* modelling, Madden et al. (2026), in their Review, focus on the use of organoid models derived from diverse bat tissues to investigate the mechanisms underlying zoonotic disease. Given the vast diversity of bat species, studying these animals and their immune responses in laboratory settings is often challenging. Therefore, bat organoids provide a valuable complementary approach to study their unique immune responses to zoonotic disease that may reveal therapeutic opportunities for patients.

In their At a Glance poster article, Tu et al. (2026) discuss androgenic alopecia, a common hair loss disorder for which treatment options remain limited owing to the lack of physiologically relevant drug-testing models that accurately predict human responses. The authors examine the roles of both animal and *in vitro* models, exploring how these approaches complement one another, and highlight potential strategies to improve their predictive value in future research.

## Modelling inaccessible disease tissue

iPSC-derived systems are also immensely useful for investigating diseases that affect tissues with limited accessibility, such as the human inner ear. Lucassen et al. (2026) developed iPSC-derived inner ear organoids to investigate drug-induced hearing loss. Using cisplatin and gentamicin, the authors recapitulated key cellular features of ototoxicity, and, intriguingly, with prolonged culture, the inner-ear organoids exhibited partial recovery, revealing an unexpected degree of developmental plasticity. This study positions inner ear organoids as powerful tools for toxicity screening and also for identifying regenerative strategies in sensory disorders.

Another disease tissue that has limited access is early-stage endometriosis, as diagnosis is often delayed. To combat this, in a Resources & Methods paper, Rahmana et al. (2026) developed a patient-derived co-culture system of endometriosis. The authors established the model with a layer of human peritoneal fibroblasts and a layer of donor-matched peritoneal mesothelial cells using an extracellular matrix scaffold and then introduced endometrial epithelial organoids, which adhered onto the layer model. This model mimicked early endometriotic lesion formation, enabling the study of lesion initiation and progression. This platform, therefore, opens the door to identifying new therapeutic targets and testing interventions in a controlled, patient-specific context.

## Recapitulating core disease tissue components and microenvironment

In many disease contexts, specific components of the diseased tissue or its environment need to be recapitulated to create a meaningful *in vitro* model system. In the lung, for instance, the epithelium is exposed to the air, albeit protected by a mucous layer. Therefore, in their study focused on cystic fibrosis, Boboltz et al. (2026) cultured human airway epithelial cells at air–liquid interface. The authors use this *in vitro* model to examine the pathological role of myeloperoxidase (MPO), a neutrophil-derived enzyme that has been correlatively linked to cystic fibrosis severity. They show that MPO directly alters mucus viscosity and composition to impair its clearance from the lung epithelium, and treatment with the reducing agent N-acetyl cysteine partially restores function. Targeting MPO or its downstream biochemical effects could, therefore, represent a therapeutic strategy not only for cystic fibrosis but also for related respiratory diseases, such as bronchiectasis.

In their At a Glance poster article, Kan et al. (2026) discuss another component critical for modelling both healthy and diseased tissues: the vasculature. Although organoid and organ-on-a-chip approaches have significantly developed over the past two decades, generating tissue models with a fully functional vascular system remains a challenge. The authors outline the technical aspects of existing approaches, evaluate their respective advantages and limitations, and provide guidance on their application, as well as perspectives on future research directions.

The tumour microenvironment plays a pivotal role in shaping disease progression and treatment response. In chronic lymphocytic leukaemia (CLL), tumour cells circulate between the blood, bone marrow and lymphoid organs, including the lymph node (LN), which is instrumental for enhancing their survival, proliferation and drug resistance. A study from Belloni et al. (2026) addresses a longstanding gap by developing a 3D LN model that recreates this niche using a gelatin scaffold and a spinning clinorotator bioreactor seeded with fibroblasts and endothelial cells. The model reproduces key *in vivo* features and, critically, reveals differential drug responses, with CLL cells in the LN-like environment exhibiting greater resistance to the BCL-2 inhibitor venetoclax than those in bone marrow-like conditions, while response to the BTK inhibitor ibrutinib remains similar across niches. These findings underscore the importance of tissue context in therapeutic efficacy, with the 3D LN model offering a predictive platform for preclinical testing.

In a similar vein, in a Perspective article, Okamoto and Takebe (2026) present a new framework for interrogating disease mechanisms in multi-tissue organoid models. They highlight how some disease mechanisms occur at the interface of two or more tissue elements, and a synergistic and interdependent decline can occur in these tissues, resulting in the pathological outcome. This Perspective highlights the importance of carefully constructing biological complexity in multi-tissue organoid models and also outlines a simple workflow that can untangle interdependent disease processes.

## Integrating innovative *in vitro* models with advancing technology

In parallel to the progress made in *in vitro* modelling, there are many advances in technologies that can be integrated with these systems. In an At a Glance poster article, Marco-Rius et al. (2026) illustrate how sophisticated organ-on-a-chip models can be used with hyperpolarised magnetic resonance spectrometry to dynamically analyse metabolites for the establishment of new biomarkers of disease and to track responses to drug treatments. Additionally, in a Review, Guragain et al. (2026) explore how innovative model systems and technologies, such as high-resolution optical mapping and optogenetic stimulation, are helping researchers overcome challenges with the transplantation of human iPSC-derived cardiomyocytes to treat myocardial infarction.

## The power of reductionist models

While many researchers are boldly reconstructing complex human biology *in vitro*, in some instances, more simplified, reductionist *in vitro* models are favoured. In our interview with Hans Clevers (Clevers, 2026), he advocates the use of simple and reproducible organoid models, especially for drug development, as more complex systems introduce more variables. These systems reliably test drugs and are important precursors for animal model pre-clinical testing.

A similar view is shared by Greulich (2026) in his Perspective, which advocates the use of mathematical and computational modelling, not merely for data fitting but as a means to translate qualitative biological ideas into quantitative, testable hypotheses. A recurring theme of the article is that experiments should be designed with the intention of falsifying rather than confirming the hypotheses, and that models should remain as simple as possible, as excessive complexity increases the risk of overfitting. At the end of the Perspective, the author also proposes practical steps for incorporating modelling into day-to-day biomedical research.

## Outlook

It seems, therefore, that the future of disease modelling lies in the diversity of available model systems. To address the range of scientific challenges we are faced with, we must harness the power of a variety of *in vitro* models that differ in biological complexity, alongside animal models. In this Special Issue, we showcase a breadth of *in vitro* systems, all carefully designed in response to specific technical limitations or scientific questions. These models can recapitulate inaccessible stages of disease and tissue types or critical architectural and microenvironmental components of diseased tissue. This is crucial for understanding fundamental mechanisms of disease, accelerating drug development pipelines and enabling customised approaches tailored to individual patients.
